# Rosin Solution in Root Filling

**Published:** 1914-11-15

**Authors:** J. R. Callahan


### ROSIN SOLUTION IN ROOT FILLING.

BY J. R. CALLAHAN, D.D.S.

The following is an abstract of a paper read by Dr. Callahan before the Rehwinkle Dental Society of Chillicothe, and is printed in full in the Dental Summary for October, 1914.

℞ Rosin ............................... gr. xij

Chloroform ............................. ʒiij

Make into a solution.

The rosin that is best adapted to dental uses that I have been able to find is that prepared by Bernardel for the use of the violinist, a French preparation very near the color of dentin. The formula as given above makes a very thin solution. It required a long time for me to realize the advantage in the use of a thin solution. A thick mixture will not penetrate the tubules, nor does it give up enough chloroform to dissolve the gutta-percha.

It is my desire to discuss the use of a solution of rosin in chloroform in our treatment of the class of cases that the three statements suggest.

*First*—That thermal changes due to the presence of large metallic fillings in tooth cavities excite conditions that cause the untimely death of many tooth pulps.

*Second*—That pulp capping has ceased to be a regular procedure in many offices.

*Third*—That a thoroughly satisfactory root-canal filling has not yet been proven.

It is not likely that in the preparation of cavities we *always* remove all of the affected dentin. In deep-seated cavities it is not advisable. In spite of the application of strong antiseptic agents recurrent decay may develop, and toxins finally reach the pulp.

If the remaining traces or thin layers of decayed dentin can be thoroughly dehydrated, the application of rosin solution may be of great service.

First, rosin being more or less a non-conductor, it reduces the shock of thermal changes, thereby lessening the tendency to secondary growths or deposits within the pulp chamber that are so noticeable under large metallic fillings, especially under large gold inlays.

We are taught that the decalcified dentin that is to be found just in advance of the micro-organisms in carious dentin furnishes food for the invading host. If the remaining decalcified dentin be saturated with rosin, I imagine the cost of living in that region will become prohibitive. However, if the rosin solution reaches the farthest boundaries of the decalcified dentin through the infected area, then the micro-organisms within the tubuli will bave been engulfed within the rosin solution, and unless the bacteria are able to liquefy the rosin, they will be forever inhibited from further activity, be they aerobic or anaerobic, in active or spore form. I need only mention the antiseptic properties of the chloroform.

This you will admit would be a very desirable condition in which to have a layer of decayed or decalcified dentin over the pulp. where the removal of the layer of decay would mean the exposure of the pulp.

The most satisfactory results that I have had in capping pulps has been to flow a rosin solution over the exposure, evaporating the chloroform with warm air, then cause a very thin cement to *flow* over the floor of the cavity and the thin coat of rosin and allow it to harden, being very careful to avoid pressure of any kind on the cement until quite hard.

This practice has been confined to quite small and recent exposures. Not the least satisfactory use of the rosin solution is after more or less thorough drying of the cavity and application of the rosin prior to the insertion of gutta-percha fillings either as a temporary or permanent filling.

On the removal of a temporary stopping of this nature that has been in place a week or a month, the decayed dentin that may have for any reason been left in the cavity will be found noticeably tough and hard and dry, due to the presence of the rosin, and the sensibility of the dentin will be materially less, showing that the dentin has been free from the irritating effects of acids, or, in other words, the fibrils have been in a state of comparative rest. And after all is said, the chief function of the surgeon is to remove the irritant and place the affected region at rest to the end that nature may perform a cure.

No root canal is properly prepared for filling unless a fine paper canal dryer, as furnished us by the dealers, can be passed to or near the apical foramen.

In a devitalized tooth we are again dealing with infected dentin or with dentin in which the tubules will soon be filled with micro-organisms, unless they be tightly sealed with a stable and compatible substance.

If possible, is it desirable or necessary that the tubuli be sealed?

Dr. Herman Prinz says: "If the canal is not filled perfectly, serum will seep into it from the apical tissues. The serum furnishes nutrient material for the micro-organisms present in the tubuli of a primarily infected root canal."

In an incipiently infected root canal, the dentinal tubuli and the small foramina offer ready hiding places for various forms of pathogenic bacteria.

After exhausting the nutrient material the bacteria become attenuated or they assume restive forms. If the tubuli and the foramina are tightly sealed, these enclosed bacteria must necessarily remain permanently confined in their lodging places, while, if the root-canal filling leaks, the seepage of serum furnishes fresh material which offers excellent opportunity for their renewed activities.

We have the testimony of several investigators to the effect that it is possible to sterilize the root canal proper, but it is an impossibility to sterilize infected dentin of a tooth while it remains in the mouth.

The microscope and the culture media have shown us conclusively that we have been, and are now, leaving enormous numbers of micro-organisms within the body with a more or less available route open to the circulatory system where they may reach any part of the body carrying destruction to those organs or parts that may offer the most attractive lodging place.

A most significant fact must be borne in mind in regard to the devitalized dentin. We have no blood current to assist in the struggle. The dentin has absolutely no power even to assist in repair. No granulation or scar tissue—nothing but an inert tubular mass infected by millions of toxin-producing micro-organisms. We must make of this infected tubular mass an inert harmless and stable body, including the effective closing of the numerous foramina, to the end that nature may be able to envelop the root mass in a healthy and vigorous peridental membrane that the tooth may serve its several useful purposes for a number of years.

Most of us have at one time or another shared in the opinion that what the root canal might be filled with mattered but little.

The radiograph in the hands of the advanced dental practitioners has brought to light evidence sufficient to prove to the dullest of comprehension the fallacy of such an opinion. It does matter as to the material; it does matter as to the manner of placing the material in the canal. The matter of prime importance being the sealing of the more or less numerous foramina, and, as we have no assurance that all the foramina in a given root canal are located near the apex it becomes our duty to seal the whole length of each canal with a material that will search out and seal minute canals or openings that, owing to physical conditions, we are unable to see.

The technic of the rosin-gutta-percha root filling is simple, easy, quick, and sure to seal all tubuli and foramina that are open.

The root canal should be the general shape of the paper root-canal driers as furnished us by the dealers, and the mouth of each canal should have a decided cone shape. This will facilitate the placing of agents or instruments to or near the apical foramen. The first step then is the complete dehydration of the dentin, using acetone as advised by Dr. Prinz, as the dehydrating agent. After flooding the canal with acetone, use the paper points liberally until the canal is entirely free of moisture. Follow this with warm air. Then hold a warm wire in the canal for a minute or two, being careful that the wire is not hot enough to scar any part of the canal.

Right here is where many root-canal operations fail. The canals and tubuli must be as dry as it is possible to make them, bearing in mind that it is possible to do damage by overheating the root.

Now flood the dry root canal with the thin rosin solution, pumping it in with a wisp of cotton on a broach. When the canal is full of the solution, pass a fine wire or broach to the end of the canal. Work out all the air that may be trapped therein. This is of vital importance.

Select a gutta-percha cone that wil reach to or near the end of the canal, holding the cone with a fine foil carrier, and pass the cone carefully and surely about half way into the canal, pumping the cone up and down in the canal usually from forty to sixty times, and, as it dissolves in the chloroform, advancing the cone farther toward the apex.

The pumping motion forces the rosin solution farther into every opening. The chloroform at the same time dissolves the periphery of the gutta-percha cone which, becoming more and more attenuated, slips farther toward the apex, surrounding itself with a mixture of gutta-percha and rosin. The rosins seals the tubuli and at the same time causes the gutta-ercha to stick tight to the pulp wall and makes the gutta-percha more stable and proof against the action of body fluids or substances.

If this does not leave the large end of the gutta-percha cone at or near the end of the canal, place a small cone alongside or on the first one; then, with cold steel plugger points that will go into the canals, gently pack the mass into the canal, using warm air to soften the protruding gutta-percha if necessary.

This packing forces the semi-fluid (chloro-percha and rosin) into unknown canals and pockets, and at the same time brings the surplus chloro-percha to the mouth of the canal, where it may be taken up with absorbent rolls or cotton.

In multi-rooted teeth complete the filling of each individual canal before starting another.

Rub the steel plugger points on paraffin cake to prevent the partially-dissolved gutta-percha from adhering to the instrument. The pulp chamber is to be filled with one of the cements.

You may ask: "Do you succeed in filling *all* canals and tubuli to the farthest extremity?" No; only those that are open and dry to the farthest extremity.

Are we likely to have inflammation in the periapical region following the closure of root canals in this manner?

The probability of inflammatory conditions in all cases depends upon the ability of the operator to read the pathological signs of each individual case and his skill and delicacy of touch in the manipulation of the various agents used.

Rosin-chloro-percha and the cone are superior to chloro percha in three ways. *First,* the rosin in chloroform penetrates deeply into the tubuli and foramina that chloro-percha will not enter at all, leaving within such tubuli or foramina, upon the disappearance of the chloroform, a more or less solid, inert, and insoluble substance that enmeshes the contents and seals the lumina of such tubuli or foramina. *Second,* the rosin and chloroform causes the gutta-percha, in whatever form it may be applied, to adhere closely to the walls of root canal or cavity. *Third,* the incorporation of the rosin in the freshly made chloro-percha makes an unshrinkable and impervious mass about the gutta-percha cone. If gutta percha and rosin be dissolved in chloroform and left in an open dish or tube to dry or solidify, the rosin will rise to the surface and harden in a crust over the gutta percha. When the mixture is made in the root canal, as has been suggested, the rosin in solution is held firmly in place in the dissolved gutta percha between the canal wall and the cone in the center.

